# The impact of active surveillance and health education on an Ebola virus disease cluster — Kono District, Sierra Leone, 2014–2015

**DOI:** 10.1186/s12879-016-1941-0

**Published:** 2016-10-27

**Authors:** Tasha Stehling-Ariza, Alexander Rosewell, Sahr A. Moiba, Brima Berthalomew Yorpie, Kai David Ndomaina, Kai Samuel Jimissa, Eva Leidman, Dingeman J. Rijken, Colin Basler, James Wood, Dumbuya Manso

**Affiliations:** 1 Centers for Disease Control and Prevention, Atlanta, GA USA; 2 School of Public Health and Community Medicine, University of New South Wales, Sydney, Australia; 3 District Health Management Team, Ministry of Health and Sanitation, Koidu, Sierra Leone; 4 International Federation of Red Cross and Red Crescent Societies, Geneva, Switzerland

**Keywords:** Ebola virus disease, Sierra Leone, Surveillance, Health education

## Abstract

**Background:**

During December 2014–February 2015, an Ebola outbreak in a village in Kono district, Sierra Leone, began following unsafe funeral practices after the death of a person later confirmed to be infected with Ebola virus. In response, disease surveillance officers and community health workers, in collaboration with local leadership and international partners, conducted 1 day of active surveillance and health education for all households in the village followed by ongoing outreach. This study investigated the impact of these interventions on the outbreak.

**Methods:**

Fifty confirmed Ebola cases were identified in the village between December 1, 2014 and February 28, 2015. Data from case investigations, treatment facility and laboratory records were analyzed to characterize the outbreak. The reproduction number (R) was estimated by fitting to the observed distribution of secondary cases. The impact of the active surveillance and health education was evaluated by comparing two outcomes before and after the day of the interventions: 1) the number of days from symptom onset to case-patient isolation or death and 2) a reported epidemiologic link to a prior Ebola case.

**Results:**

The case fatality ratio among the 50 confirmed Ebola cases was 64.0 %. Twenty-three cases occurred among females (46.0 %); the mean age was 39 years (median: 37 years; range: 5 months to 75 years). Forty-three (87.8 %) cases were linked to the index case; 30 (61.2 %) were either at the funeral of Patient 1 or had contact with him while he was ill. R was 0.93 (95 % CI: 0.15–2.3); excluding the funeral, R was 0.29 (95 % CI: 0.11–0.53). The mean number of days in the community after onset of Ebola symptoms decreased from 4.0 days (median: 3 days; 95 % CI: 3.2–4.7) before the interventions to 2.9 days (median: 2 days; 95 % CI: 1.6–4.3) afterward. An epidemiologic link was reported in 47.6 % of case investigations prior to and 100 % after the interventions.

**Conclusions:**

Initial case investigation and contact tracing were hindered by delayed reporting and under-reporting of symptomatic individuals from the community. Active surveillance and health education contributed to quicker identification of suspected cases, interrupting further transmission.

## Background

The largest known Ebola virus disease (Ebola) outbreak occurred in West Africa in 2014–15 [1]. In Sierra Leone, one of the three heavily affected countries, Ebola was first recognized in May 2014 [1] and eventually spread to all 14 districts in the country. Health officials in Kono district, located on the eastern border with Guinea, identified approximately 230 confirmed Ebola cases between December 2014 and April 2015, after which there was no evidence for ongoing transmission in Kono. Fifty of these case-patients lived or worked in one village.

Ebola virus is a filovirus (Filoviridae family) with three species known to cause large outbreaks of disease in humans; the outbreak in West Africa involved the Zaire species. Symptoms of Ebola infection include fever, headache, fatigue, muscle pain, vomiting, diarrhea, abdominal pain, and unexplained hemorrhage. The incubation period ranges 2–21 days (mean 4–10). Death or recovery usually occurs 6–16 days or 6–11 days after symptom onset, respectively. The case fatality rate of the Zaire Ebola species has been reported as 60–90 % in prior outbreaks [2, 3].

The introduction of Ebola virus into human populations likely occurs via contact with an infected animal, such as a fruit bat [2, 3]. However, during an outbreak, person-to-person transmission occurs through direct contact with the body or body fluids of an infected case, including blood, vomit, saliva, urine, feces, and semen, through broken skin or unprotected mucous membranes [2–4]. Caring for infected persons and funeral practices in which attendees have direct contact with the deceased can be particularly dangerous [3, 5].

Key strategies to control an Ebola outbreak include setting up a surveillance system to interrupt transmission and social mobilization and health education programs to promote protective behaviors and discourage high-risk behaviors in communities [3]. Active surveillance helps identify symptomatic patients rapidly so they can be isolated, in order to reduce the likelihood of transmission to others, and treated. Case investigations, as part of surveillance activities, identify potential sources of infection and contacts of suspected cases. In turn, this information can inform health education messages and interventions around high-risk behaviors in the community and facilitate monitoring of contacts for 21 days, i.e. contact tracing, allowing for their rapid isolation and treatment should symptoms develop.

### Cluster description

**Fig. 1:**
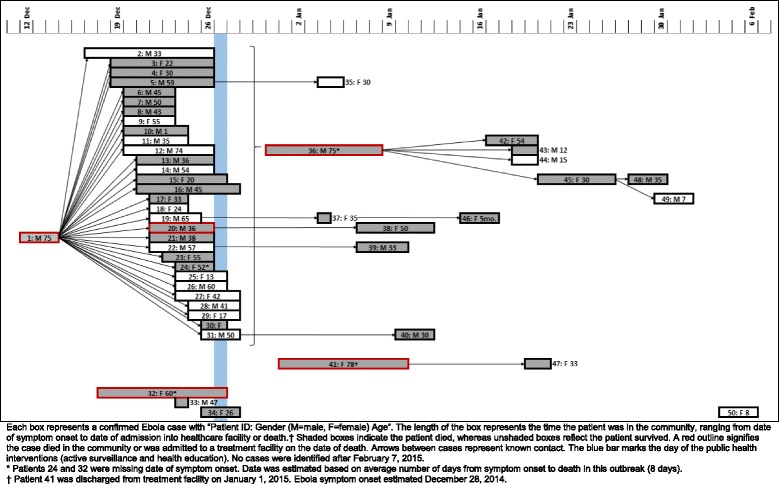
Ebola transmission and time in community after symptom onset for 50 patients with confirmed Ebola

On December 14, 2014, disease surveillance officers investigated the death of a 75 year-old taxi driver and community leader (Patient 1, the index case, Fig. 1) from a village in Kono District (population approximately 1,000 residents) not previously known to be an area of ongoing transmission. Two days prior, Patient 1 had reported to the district hospital with unknown symptoms. He was clinically diagnosed with a stroke, his second, and sent home. Within 48 h of discharge, he developed diarrhea and vomiting and died at home. The day following his death, the district burial team was informed, obtained an oral swab sample for laboratory testing (which was later confirmed to be positive), and buried the body. The team noted that the body had been washed and prepared for burial according to traditional funeral practices that were banned after the Ebola epidemic began; however, the family denied holding a funeral or touching the body. No epidemiologic link to an Ebola case, alive or deceased, was discovered. District surveillance officers identified 14 contacts of the decedent from four households for quarantine and monitoring.

On December 23, district surveillance officers investigated six suspected Ebola cases in the village where Patient 1 died. Five more suspected Ebola cases were identified during the next 2 days, followed by an additional 15 suspected Ebola cases on December 26. Initial investigations of these suspected cases did not clearly identify an epidemiologic link to Patient 1 or to any other suspected or confirmed case. Furthermore, delayed reporting of additional symptomatic individuals and contacts of suspected Ebola cases hindered public health officials’ ability to respond swiftly.

On December 27, district leaders, disease surveillance officers, and local and international partners conducted two public health interventions in the village: active surveillance to identify symptomatic individuals for isolation and treatment and health education for all residents.

Initial interviews were conducted with suspected case-patients or their proxies, e.g. spouses and adult children, to gather information about Ebola exposures. In the weeks that followed, surveillance officers made daily visits and conducted numerous follow-up interviews.

We conducted a field investigation to determine the likely sources of Ebola infection for the index case and other cases in the village, and potential opportunities for prevention of future Ebola infections.

### Objectives

This paper aims to describe the field investigation, providing insight into the role of high risk behaviors—e.g., unsafe funeral practices and care of infected persons—in driving the outbreak in this village, and evaluate the impact of active surveillance and health education on preventing further Ebola transmission.

## Methods

Ebola cases were defined according to national protocols at the time [6]. A suspected Ebola case required fever and at least three other symptoms OR fever or bleeding in someone with an epidemiological link to an Ebola case OR a sudden, unexplained death; a probable case required fever and at least three other symptoms and an epidemiological link OR a sudden, unexplained death and an epidemiological link. Cases were confirmed using reverse transcriptase polymerase chain reaction (RT-PCR) testing on blood specimens collected from patients or oral swabs collected from deceased individuals [6].

Suspected cases reported by health facilities or the general public were investigated using a nationally standardized case investigation form. Surveillance officers collected information on clinical presentation, prior medical care received for this illness, and any epidemiologic link to suspected or confirmed Ebola cases. Epidemiologic links included contact with a sick person (alive or dead), funeral attendance and/or contact with the body, travel away from home, and visiting a healthcare facility prior to symptom onset. Contact with a sick person or body was defined as touching body fluids of a case (blood, vomit, saliva, urine, feces), direct physical contact with the body (alive or dead), touching or sharing clothes, linens, or eating utensils, or sleeping, eating, or spending time in the same room or household as a case. When possible, officers interviewed the patient directly; in the case of a death investigation or when the patient was too ill to be interviewed, family members or other proxies were interviewed. Officers also identified and collected detailed data on contacts of suspected cases for monitoring and possible quarantine.

The public health interventions, active surveillance and health education, were conducted on December 27 by disease surveillance officers, community health workers, ambulance personnel, district leaders, and local and international partners. Active surveillance consisted of visiting every home in the village (approximately 200 homes) to identify symptomatic individuals. All residents of each home were asked to come outside and interviewed to determine if they were currently experiencing symptoms consistent with Ebola. Trained health education teams then began to educate residents on symptoms of Ebola, how to notify authorities about sick individuals through the national hotline, and the importance of early treatment for those who are infected. During this interaction, community health workers and surveillance officers observed the residents for signs of illness. Symptomatic individuals were isolated, interviewed using standard case investigation forms, and transported by ambulance to the Ebola holding center at the district hospital and, later, an Ebola treatment unit in another district.

Case investigation data were entered into the district Ebola database (Epi Info 7, CDC, Atlanta, GA). Information subsequently collected from laboratory testing and patient outcome data were added to the same database. Case investigators gathered additional information during follow-up visits and interviews.

Qualitative data from interviews with disease surveillance officers and data from case investigations, treatment facility records, and laboratory results for all suspected, probable, and confirmed cases of Ebola identified in the village between December 1, 2014 and February 28, 2015 were analyzed to characterize the epidemiology of the outbreak. Insufficient data were available on cases identified in October and November 2014 (four suspected and one probable) and were excluded; none were known to be associated with the outbreak described here from the limited information available. As of October 24, 2016, no cases have been identified in the village since February 2015.

The reproduction number (R) was estimated with and without Patient 1’s funeral. Estimates based on Poisson and negative binomial distributions for secondary cases were compared using the Akaike information criteria (AIC) to determine the best model fit [7]. Confidence intervals were generated using non-parametric bootstraps (1000 resampled data sets).

The impact of public health interventions was evaluated using two outcome variables: 1) the amount of time after symptom onset that a suspected case-patient remained in the community before isolation or death and 2) whether the case-patient had a known epidemiologic link. For both outcomes, we compared cases with symptom onset before and after the interventions on December 27. Time in the community was calculated as the number of days from reported onset of symptoms to either death in the community or admission to a healthcare facility. Patient 41 was in a healthcare facility at the time of reported symptom onset; her time in the community was calculated from date of discharge to her death in the community. Reporting of epidemiologic links was expected to improve with health education. Few epidemiologic links were reported during the initial case investigations, despite obvious signs of body washing and preparation; we hypothesized health education would improve willingness to report any links. Unknown links to Ebola cases from other districts also were considered fairly unlikely due to limited mobility of the village residents. All confirmed Ebola cases in the village (the entire study population) were included in the analyses; however, given the objective to compare the pre- and post-intervention outcomes, *t*-test and chi-squared statistical tests were conducted to assist in interpreting the results.

## Results

Between December 1, 2014 and February 28, 2015, a total of 50 laboratory confirmed Ebola cases (including the index case) were identified. The case fatality ratio among the 50 confirmed Ebola cases was 64.0 % (*n* = 32), of whom 15.6 % (*n* = 5) died in the community or on the day they were admitted to a healthcare facility. Twenty-three cases occurred among females (46.0 %). The mean age of the 50 Ebola case-patients was 39 years (median: 37 years; range: 5 months to 75 years), six were aged 0–14 years, 27 were aged 15–49 years, and 17 were 50 years and older. Four (8 %) cases provided healthcare either to confirmed cases in their homes, such as with family or neighbors, or in a healthcare facility.

The investigation linked 43 (87.8 %) of the 49 confirmed Ebola cases (other than Patient 1) to Patient 1, 27 (62.8 %) of whom died (Fig. 1). Of the 49 Ebola cases (other than Patient 1), 30 (61.2 %) Ebola cases were first generation, directly linked to either the funeral of Patient 1 or contact with him while he was ill. Following further investigation, disease surveillance officers determined that 24 (49.0 %) of the 49 Ebola case-patients likely were infected at the index patient’s funeral. Thirteen additional confirmed Ebola cases resulted from exposure to secondary cases, including a teacher who cared for several secondary cases (Patient 36).

Six confirmed Ebola cases were not linked to Patient 1, the index case. In two cases, (Patients 32 and 33) patients had symptom onset dates consistent with exposure to Patient 1, but could not be directly linked to Patient 1 through initial case investigations or follow-up interviews after the outbreak. One of these cases occurred in a nurse at the district hospital who had cared for at least one confirmed Ebola case-patient from another village. Another confirmed Ebola case-patient (Patient 41) was identified as having symptoms consistent with Ebola during the active surveillance, but tested negative for Ebola twice and was sent home on January 1, 2015. This patient died in the community on January 10 and was confirmed as infected with Ebola post-mortem. The patient may have become infected on December 27 while being transported in an ambulance on rough, unpaved roads for 4 h to an Ebola treatment unit with other patients later confirmed to have Ebola. She is suspected to be the source case for her child (Patient 47). The final, unlinked confirmed Ebola case (Patient 50) likely was infected through exposure to a family member from another village who had a confirmed Ebola case.

**Fig. 2:**
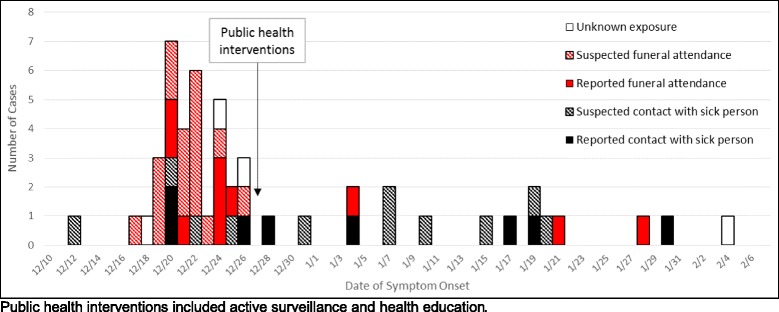
Number of confirmed Ebola cases (*N* = 50) by date of symptom onset and exposure type

Among the 50 confirmed Ebola cases, funeral attendance and unsafe funeral practices were the likely sources of infection for 27 (54.0 %) cases whereas caring for or contact with sick patients was the likely source for 19 (38.0 %) others (Fig. 2). None of the confirmed Ebola case-patients reported travel to affected areas outside the village.

Among the 46 cases with explicit links, the mean reproduction number (R) was 0.93 (95 % CI: 0.15–2.3) with a variance of 9.83 (95 % CI: 0.16–77) using a negative binomial distribution. Excluding Patient 1’s funeral event, R was 0.29 (95 % CI: 0.11–0.55), with variance 0.49 (95 % CI: 0.11–1.48). In each instance, Poisson means were similar but the fits from the negative binomial distribution were a vast improvement when the funeral case was included (AIC 45 vs 125 for Poisson) and slightly better without the funeral case (32.4 vs 34).

Not all of the 50 confirmed Ebola cases met the suspected or probable Ebola case definition. Of 32 confirmed Ebola cases with sufficient information on symptoms and potential epidemiologic links, nine (69.2 %) surviving Ebola cases and 14 (73.7 %) deceased Ebola cases met the probable or suspected Ebola case definition at the time of the initial investigation (Table 1). Of the nine who did not meet case definition, seven (87.5 %, one missing) reported not having an elevated temperature, four (44.4 %) reported fewer than three symptoms, and five (55.6 %) reported no epidemiologic link to a suspected or confirmed Ebola case.

**Table 1:** Status of patients (*N* = 32) with confirmed Ebola based on initial case investigation data^a^

Case definition at initial presentation	Survivors	Deceased	Total
	No. (%)	No. (%)	No. (%)
Insufficient information to record as suspect or probable Ebola case	4 (30.8 %)	5 (26.3 %)	9 (28.1 %)
Suspected Ebola case	6 (46.2 %)	5 (26.3 %)	11 (34.4 %)
Probable Ebola case	3 (23.1 %)	9 (47.4 %)	12 (37.5 %)
Total	13 (100 %)	19 (100 %)	32 (100 %)

^a^Among patients with complete record of symptoms and epidemiological history

Before the public health interventions, the mean number of days in the community after onset of Ebola symptoms was 4.0 days (median: 3 days; 95 % CI: 3.2–4.7). After the public health interventions, the mean days in the community decreased to 2.9 days (median: 2 days; 95 % CI: 1.6–4.3); this was not statistically different from the pre-intervention mean (*t* = −1.5, *p* = 0.15). However, when excluding Patient 41 who was sent home after testing negative for Ebola and later died of Ebola at home, the time in the community was significantly lower at 2.5 days (median: 2 days; 95 % CI: 1.4–3.7; *t* = −2.2, *p* = 0.04) after the interventions. The earlier detection of confirmed Ebola cases did not lead to improved survival, however; the case fatality ratio increased from 58.8 to 73.3 % (*X* ^2^ = 1.2, *p* = 0.27).

Follow-up interviews conducted by surveillance officers led to improved understanding of the outbreak and data collection as survivors and family members often provided information that had been withheld during earlier interviews. For example, it was not until surveillance officers conducted follow-up interviews with a work colleague and confirmed Ebola case that they learned how Patient 1 likely was infected. In early December 2014, Patient 1 drove a sick family member from a neighboring village to the district hospital; vomitus from the sick passenger got on both Patient 1 and his taxi. Although Patient 1 did not inform his family, he insisted they keep their distance from him. After his death on December 14, friends and relatives held a secret funeral at night, washing and preparing his body for burial, before calling for the district safe-burial team. Though the details of this funeral are unknown, touching and/or kissing the body of the deceased is common and, like washing the body, increases the likelihood of direct contact with the deceased’s body fluids and the virus.

Survivors and family members also provided more information during initial investigations after the public health interventions. During the initial case investigation of Patient 1, only seven (23.3 %) of the 30 confirmed Ebola cases likely infected by Patient 1 were identified as contacts. Before the interventions, 47.6 % of initial case investigations (10 of 21 with data available) identified an epidemiologic link to a prior Ebola case (as reported by the patients or their proxies). After the interventions, 100 % (8 of 8 with data available) of case investigations identified epidemiologic links. This difference was statistically significant (*X* ^2^ = 7.4, *p* < 0.01).

## Discussion

The 2014–2015 Ebola epidemic in West Africa was the largest ever. In a village in eastern Kono District, Sierra Leone, the delayed confirmation of Ebola in a patient followed by high-risk behaviors (unsafe funeral practices and caring for ill individuals without adequate personal protective equipment) led to an Ebola outbreak with 43 additional confirmed Ebola cases and 27 deaths.

The active surveillance and health education implemented by health authorities and partners in the village appeared to contribute to the reduced time from onset of disease to reporting of suspected cases and health facility admission and improved ability of disease surveillance officers to link suspected and confirmed Ebola cases during initial case investigations. Based on our experience in the field, we believe that these changes were at least partially due to improved trust in health authorities; however, the impact of the interventions cannot be distinguished from improved cooperation of patients and their proxies due to the trauma of witnessing their family, friends, or neighbors becoming ill with Ebola. Overall, the decrease in mean time in the community after Ebola symptoms developed likely positively contributed to a more rapid reduction in the spread of Ebola in the community (Fig. 2). The survival of confirmed Ebola cases decreased after the public health interventions. This may be due to chance, considering the small number of cases, but also may reflect the limited medical care available in treatment facilities that were receiving patients from multiple, concurrent outbreaks.

The R in this Ebola outbreak was 0.93 overall, consistent with investigations of other outbreaks. A study of 15 Ebola outbreaks in Liberia found *R* = 1.7; R was reduced to 0.1 after the implementation of immediate isolation or transfer of symptomatic persons to Ebola treatment units, contact identification and monitoring [8]. In this outbreak, all but five confirmed cases were transferred to the Ebola holding center or treatment unit, the time to transfer decreased, and contact identification improved over time. When Patient 1’s funeral event was removed from the analysis, the R decreased from 0.93 to 0.29, likely reflecting the effect of active surveillance and quicker reporting of ill residents to health authorities following the Ebola transmission from the funeral. In general, the reproduction number varies across cultural, geographic settings, and at different time points in an outbreak [9]; therefore some variation from findings in Liberia is expected.

The accurate identification of suspected Ebola cases is crucial for early detection, improved patient outcomes, and limiting community transmission [9, 10]. However new diseases present diagnostic challenges, particularly early in outbreaks when clinician familiarity may be limited; additionally, unusual clinical presentation or comorbid conditions may make diagnosis more difficult. Timely distribution of information to health facilities could help clinicians to accurately diagnose new diseases, particularly during outbreaks of high-consequence pathogens such as Ebola. Also helpful is provision to patients and contacts of clear materials upon discharge from health facilities that describe signs and symptoms of the disease and steps to take should symptoms of disease develop. Wider distribution of health education materials and messages throughout communities could improve identification of additional cases.

We documented several instances in which critical information was withheld by patients or their proxies during initial investigation, such as the fact that a funeral had been conducted for Patient 1, likely exacerbating transmission in this outbreak. This denial likely was due to the national legislation banning such practices and may have affected contact tracing and the early identification and isolation of suspected cases, cornerstones of Ebola prevention and control activities [11]. Simple messages and materials educating the general public on the importance of avoiding high-risk behaviors, even when Ebola is not suspected, may promote cooperation with health authorities during case investigations and other public health activities.

Nine patients who were ultimately confirmed as Ebola cases (and who had a complete medical record) apparently did not meet the Ebola suspected or probable case definition upon initial presentation due to failure to identify or report key Ebola symptoms or a history of contact with an Ebola patient. These cases highlight the known challenge to early case identification using current case definitions [12] particularly when case investigators do not have clinical training and patients are not evaluated by a clinician at the time of the case investigation. These individuals may increase the risk of infection among their caregivers and healthcare workers if they are not recognized as suspected cases. Health promotion messages discouraging the public from caring for sick family members, whenever possible, during an outbreak and early infection control interventions at health facilities are warranted. Alternative case definitions for Ebola, with consideration for severely ill patients from areas with high transmission, should be investigated for use in future outbreaks.

## Conclusions

A single missed diagnosis and unsafe funeral resulted in a large Ebola outbreak in a village in Kono with 43 confirmed cases and 27 deaths over 8 weeks. Active surveillance and community health education were associated with a decrease in days from Ebola onset to health facility admission and may be considered for other similar outbreaks. Timely, clear materials and messages to educate the public on signs and symptoms of disease, steps to report potential cases, and the importance of avoiding and reporting high-risk behaviors are essential for early case identification, isolation, and reduced transmission in the community.

## Abbreviations

CDC: Centers for Disease Control and Prevention
CI: Confidence interval
R: Reproduction number
RT-PCR: Reverse transcriptase polymerase chain reaction
